# Potential immune evasion of the severe acute respiratory syndrome coronavirus 2 Omicron variants

**DOI:** 10.3389/fimmu.2024.1339660

**Published:** 2024-02-23

**Authors:** Luyi Chen, Ying He, Hongye Liu, Yongjun Shang, Guoning Guo

**Affiliations:** ^1^ Chongqing Nankai Secondary School, Chongqing, China; ^2^ Department of Orthopedics, Kweichow MouTai Hospital, Renhuai, Zunyi, Guizhou, China

**Keywords:** SARS-CoV-2, Omicron variant, hybrid immunity, original antigenic sin, immune evasion

## Abstract

Coronavirus disease 2019 (COVID-19), which is caused by the novel severe acute respiratory syndrome coronavirus 2 (SARS-CoV-2), has caused a global pandemic. The Omicron variant (B.1.1.529) was first discovered in November 2021 in specimens collected from Botswana, South Africa. Omicron has become the dominant variant worldwide, and several sublineages or subvariants have been identified recently. Compared to those of other mutants, the Omicron variant has the most highly expressed amino acid mutations, with almost 60 mutations throughout the genome, most of which are in the spike (S) protein, especially in the receptor-binding domain (RBD). These mutations increase the binding affinity of Omicron variants for the ACE2 receptor, and Omicron variants may also lead to immune escape. Despite causing milder symptoms, epidemiological evidence suggests that Omicron variants have exceptionally higher transmissibility, higher rates of reinfection and greater spread than the prototype strain as well as other preceding variants. Additionally, overwhelming amounts of data suggest that the levels of specific neutralization antibodies against Omicron variants decrease in most vaccinated populations, although CD4^+^ and CD8^+^ T-cell responses are maintained. Therefore, the mechanisms underlying Omicron variant evasion are still unclear. In this review, we surveyed the current epidemic status and potential immune escape mechanisms of Omicron variants. Especially, we focused on the potential roles of viral epitope mutations, antigenic drift, hybrid immunity, and “original antigenic sin” in mediating immune evasion. These insights might supply more valuable concise information for us to understand the spreading of Omicron variants.

## 1 Introduction

Since its initial emergence in Wuhan at the end of 2019, severe acute respiratory syndrome coronavirus 2 (SARS-CoV-2) has caused more than 773 million cases of COVID-19 (coronavirus disease 2019 infected by SARS-CoV-2) and more than 6 million deaths as of January 10, 2024 (1). Theoretically, all viruses can mutate by evading host immune system surveillance and causing infection or reinfection. Multiple mutations in SARS-CoV-2 lead to the emergence of many variant strains, including Alpha, Beta, Gamma, Delta, Kappa and Omicron (2). These variants differ from each other in terms of transmissibility, the capacity to penetrate somatic cells, and resistance to immune system responses, therefore, COVID-19 has remained one of the most serious public health problems worldwide (3).

The Omicron variant, first identified in Botswana, a country in South Africa, at the end of 2021, is rapidly spreading through more than 170 countries due to its exceptionally strong infectivity (4). The Omicron variant, and several sublineages, including BA.1, BA.2, BA.1, BA.2.12.1, BA.2.3, BA.2.9, BA.3, BA.4, and BA.5 have been reported owing to the extremely high rate of transmission. Omicron-helixes have numerous new mutations in the receptor binding domain (RBD) of the S protein that strongly enhance the binding affinity between the RBD and the hACE2 complex (5). Therefore, the elderly individuals have the most obvious suffering from Omicron infection, which has led to a high mortality rate during the epidemic (6). Experimental assessments are performed to understand antibody neutralization and immune responses against Omicron variants, however, Omicron spreads faster, suggesting that Omicron can escape the first line of defense provided by vaccines (7). The possibility of immune escape mechanisms, transmission and fitness on the main Omicron variants have been summarized in some previous literatures (8, 9).

In this review, we firstly introduce the epidemic properties including transmissibility and pathogenicity of Omicron and its new subvariants in a more comprehensive and timely manner. We then focus on exploring the underlying mechanisms including viral epitope mutations, antigenic drift, hybrid immunity, and the “original antigenic sin” that might be involved in mediating Omitted immune evasion. These insights may contribute to stopping Omicron transmission and provide relevant theories and countermeasures for ending COVID‐19 epidemics.

## 2 Sublineages of Omicron variants

The Delta wave gradually faded in most countries but was replaced by a new variant, Omicron, and has become the fourth peak driving the epidemic worldwide (10). Compared to earlier variants such as Alpha, Beta, Gamma, and Delta, the Omicron variant has undergone substantial changes in its amino acid sequence. The WHO has reported that Omicron variants possess seven subvariants, including BA.1 (B.1.1.529.1), BA.1.1, BA.2 (B.1.1.529.2), BA.3 (B.1.1.529.3), BA.2.12.1, BA.4, BA.5 and XBB (11). Importantly, the Omicron BA.2.12.1, BA.4, and BA.5 subvariants are phylogenetically independent of the BA.2 evolutionary branch. Complete sequencing can detect all these lineages, and the BA.2 lineage has been called ‘Stealth Omicron’ because it significantly differs from the prototype (Wuhan strain) (12). The Omicron BA.4 and BA.5 subvariants are currently at low endemic levels globally. The rapid spread of new subvariants of Omicron, such as XBB.1.5, has led to a rapid increase in prevalence, suggesting its potential for further global pandemics (13, 14).

## 3 The amino acid mutations of the Omicron variants

Compared to the Wuhan-Hu-1 strain (NC 045512.2), genome-wide annotations showed there are 16,954 mutations in the SARS-CoV-2 genome, and the D614G and N501Y are the top two deleterious mutations in the S-protein on a global scale (15). The spike protein of the Omicron variant harboured 876 mutations, including 443 deleterious mutations. Especially, mutations affecting spike proteins are mostly found in RBD regions for Omicron (16). A total of 42 mutations are present in the Omicron variant spike (S) protein, six are deletion mutations, 30 are substitutional, and one is an insertional mutation (17). The amino acid mutations in the S-protein of different SARS-CoV-2 variants are summarized in **Table 1**. Most of the mutations are located in the *S*-gene and are identical to the Delta and Alpha variants (A67V, del69-70, T95I, del142-144, G446S, S477N, T478K, E484A, Y145D, del211, L212I, S373P, S375F, K417N, N679K, P681H, N764K, D796Y, N440K, Q493R, G587S, Q498R, N501Y, Y505H, T547K, D614G, H655Y, N856K, Q954H, ins214EPE, G339D, S371L, N969K, and L981F) (24). There are 37 mutations in the S-protein of the BA.1 mutant, while the BA.2 mutant has 31 mutations and the BA.3 mutant has 33 mutations in this region. It has been reported that the RBD of Omicron variants has 15 mutation sites, including K417N, S447N, T478K, N440K, G446S, S371L, S373P, S375F, Q493R, G496S, E484A, N501Y, G339D, Q498R, and Y505H, these mutations strongly impact disease transmission, pathogenesis, and vaccine efficacy (25).

**Table 1:** Comparatively summarized the amino acid mutations in S-protein of different SARS-CoV-2 variants.

WHO Sublineage	Pango pedigree	The aminol acid mutants within S-protein
Prototype		/
D614G		D614G
Alpha	B.1.1.7	del69-70HV, del144Y, N501Y, A570D, D614G, P681H, T716I, S982A, D1118H
Beta	B.1.351	L18F, D80A, D215G, del242-244LAL, R246I, K417N, E484K, N501Y, D614G, A701V
Gamma	P.1	L18F, T20N, P26S, D138Y, R190S, K417T, E484K, N501Y, D614G, H655Y, T1027I, V1176F
Eta	B.1.525	Q52R, A67V, H69del, V70del, Y144del, D614G, Q677H, F888L
Iota	B.1.526	L5F, T95I, D253G, E484K, D614G, A701V
Kappa	B.1.617.1	G142D, E154K, L452R, E484Q, D614G, P681R, Q1071H, H1101D
Lambda	C.37	G75V, T76I, DEL246/252, D253N, L452Q, F490S, D614G, T859N
Mu	B.1.621	T95I, Y144S, Y145N, R346K, E484K, N501Y, D614G, P681H, D950N
	B.1.640.2	P9L, E96Q, CNDPFLGVY136-144Del, R190S, D215H, R346S, N394S, Y449N, E484K, F490S, N501Y, D614G, P681H, T859N, D1139H
Delta	B.1.617.2	T19R, G142D, E156del, F157del, R158G, L452R, T478K, D614G, P681R, D950N
Omicron	BA.1	A67V, H69del, V70del, T95I, G142D, V143del, Y144del, Y145del, N211del, L212I, ins214EPE, G339D, S371L, S373P, S375F, K417N, N440K, G446S, S477N, T478K, E484A, Q493R, G496S, Q498R, N501Y, Y505H, T547K, D614G, H655Y, N679K, P681H,N764K, D796Y, N856K, Q954H, N969K, L981F
	BA.3	A67V, HV69-70Del, T95I, G142D, VYY143-145Del, N211Del, L212I, G339D, S371F, S373P, S375F, D405N, K417N, N440K, G446S, S477N, T478K, E484A, Q493R, Q498R, N501Y, Y505H, D614G, H655Y, N679K, P681H, N764K, D796Y, Q954H, N969K
	BA.2	T19I, L24del, P25del, P26del, A27S, G142D, V213G, G339D, S371F, S373P, S375F, T376A, D405N, R408S, K417N, N440K, S477N, T478K, E484A, Q493R, Q498R, N501Y, Y505H、D614G, H655Y, N679K, P681H, N764K, D796Y, Q954H, N969K
	BA.2.12.1	T19I, L24del, P25del, P26del, A27S, G142D, V213G, G339D, S371F, S373P, S375F, T376A, D405N, R408S, K417N, N440K、L452Q, S477N, T478K, E484A, Q493R, Q498R, N501Y, Y505H, D614G, H655Y, N679K, P681H, S704L, N764K, D796Y, Q954H, N969K
	BA.2.75	T19I, 23insLPP, L24del, P25del, P26del, A27S, G142D, K147E, W152R, F157L, I210V, V213G, G257S, D339H, S371F, S373P, S375F, T376A, D405N, R408S, K417N, N440K, G446S, N460K, S477N, T478K, E484A, Q498R, N501Y, Y505H, D614G, H655Y, N679K, P681H, N764K, D796Y, Q954H, N969K
	BA.2.76	T19I, L24del, P25del, P26del, A27S, G142D, V213G, Y248N, G339D, S371F, R346T, S373P、S375F, T376A, D405N, R408S, K417N, S477N, T478K, E484A, Q493R, Q498R, N501Y, Y505H, D614G, H655Y, N679K, P681H, N764K, D796Y, Q954H, N969K
	XBB	T19I, LPP24-26del, A27S, V83A, G142D, Y145del, H146Q, Q183E, V213E, G339H, R346T, L368I, S371F, S373P, S375F, T376A, D405N, R408S, K417N, N440K, V445P, G446S, N460K, S477N, T478K, E484A, F486S, F490S, Q498R, N501Y, Y505H, D614G, H655Y, N679K, P681H, N764K, D796Y, Q954H, N969K
	XBB.1.5	T19I, LPP24-26del, A27S, V83A, G142D, Y145del, H146Q, Q183E, V213E, G252V, G339H, R346T, L368I, S371F, S373P, S375F, T376A, D405N, R408S、K417N, N440K, V445P, G446S, N460K, S477N, T478K, E484A, F486P, F490S, Q498R, N501Y, Y505H、D614G, H655Y, N679K, P681H, N764K, D796Y, Q954H, N969K
	CH.1.1	T19I, A27S, G142D, K147E, W152R, F157L, I210V, V213G, G257S, D339H, R346T, S371F, S373P, S375F, T376A, D405N, R408S, K417N, N440K, K444T, G446S, L452R, N460K, S477N, T478K, E484A, F486S, Q498R, N501Y, Y505H、D614G H655Y, N679K, P681H, N764K, D796Y, Q954H, N969K

* This table represents a detailed comparative overview of the amino acid mutation in S protein from most studied variants of SARS-CoV-2 (18–23).

T478K, E484A, Q493R, and N501Y are considered the most crucial mutations to the Omicron variants. It seems that Q498R, N501Y, Q493R, Q498R, E484A, T478K, and S477N, especially the combination of Q498R and N501Y at the RBD/ACE2 interface, might enhance Omicron’s binding affinity with the hACE2 receptor, thereby increasing Omicron infectivity (26). Importantly, Q498R is reportedly responsible for enhancing the binding of the Omic RBD to mouse mACE2 (27). Interestingly, the S477N, T478K, and E484A mutations (Omicron variant BA.1 and BA.2) near the antibody-binding region result in altered local conformations and hydrophobic microenvironments of the S protein, rendering them unrecognizable by most antibodies; these mutations are suspected to be associated with immune escape and potentially affect the virus’s behavior (28). Moreover, structural analysis of the Omicron variant spike protein-hACE2 complex revealed that the Q493R, G496S, and Q498R mutations could compensate for the weakened affinity caused by K417N (29). Additionally, there are three mutations (H655Y, N679K, P681H) near the S1/S2 Furin protease cleavage site, which have been hypothesized to facilitate virus entry into host cells and thereafter enhance Omicron’s transmissibility and pathogenicity (30).

**Figure 1:**
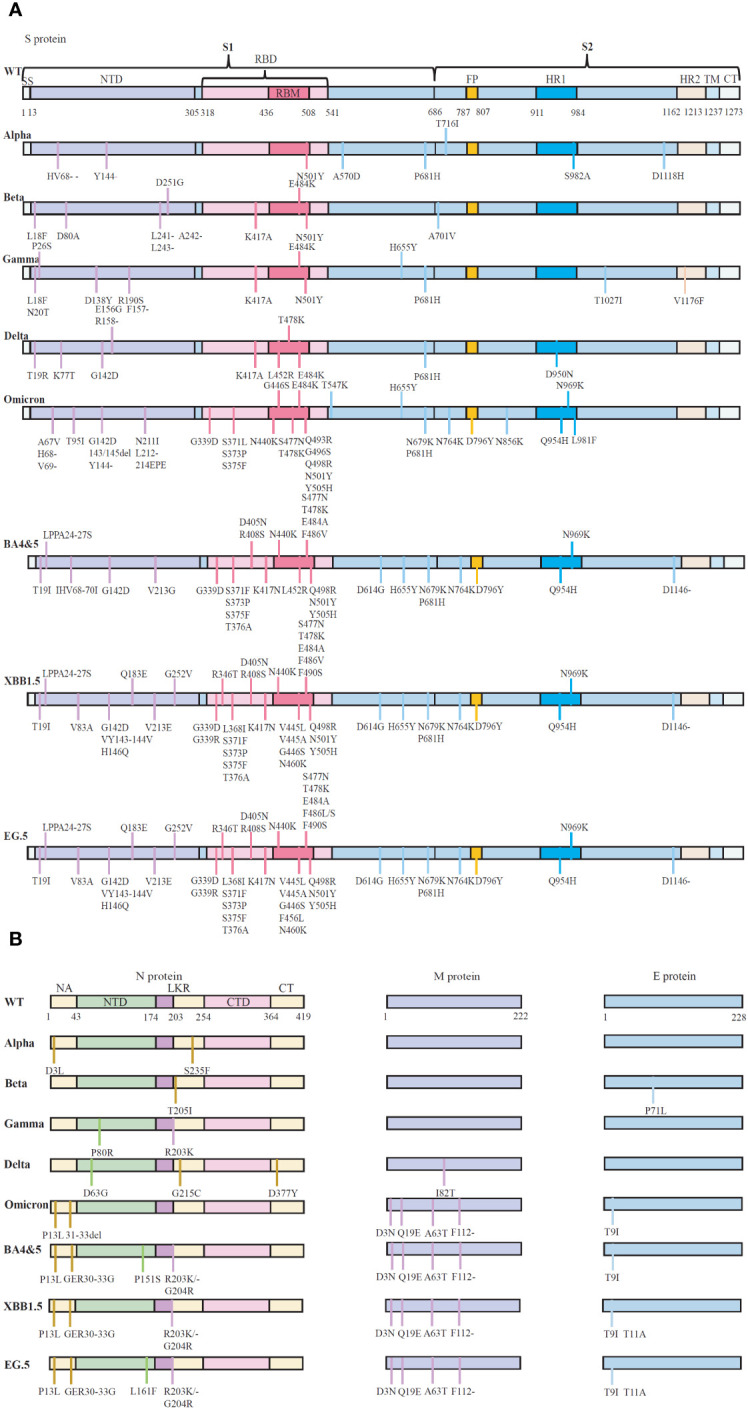
The schematic representation of the mutations in protein of five SARS-CoV-2 variants of concern (VOCs). **(A)** Reported essential mutations in the S-glycoprotein of SARS-CoV-2 variants. **(B)** Reported critical mutations in the N, M and E proteins of SARS-CoV-2 variants. Data are from WHO (Coronavirus disease (COVID-19): Variants of SARS-COV-2, https://www.who.int/news-room/). NTD, N-terminal domain; CTD, C-terminal domain; LKR, Central linker region; SP, Signal peptide; RBD, Receptor-binding structural domain; RBM, Receptor-binding motif; FP, Fusion peptide; HR1, Heptapeptide repeat 1; HR2, Heptapeptide repeat 2; TM, Transmembrane structural domain; CT, Cytoplasmic tail. WT, Wuhan-Hu-1 strain.

In addition to the S-protein, the Omicron variant contains four mutations, three deletions and one insertion (A67V, del69/70, T95I, G142D, del143/145, N211I, del212, and ins214EPE) in the nucleocapsid (N) protein (31). It seems that R203K/G204R mutations might promote viral RNA expression, possibly supporting ribonucleocapsid assembly and increasing cell permeability, thus potentially increasing virulence (32). Omicron also has one mutation in the E protein and three mutations in the M protein (31). Consequently, Omicron has emerged as a superstrain with significantly enhanced transmissibility and pathogenic potential compared to other Omicron strains (**Figure 1**).

## 4 The recent epidemic caused by Omicron variants

The symptoms of Omicron variant infected patients were similar to those of previous other variants infected patients, and included runny nose (76.5%), headache (74.4%), sore throat (70.5%), sneezing (63.5%), persistent cough (49.8%), and hoarseness (42.6%). Additionally, some Omicron variant infected patients also experience eye pain, dizziness, and high fever (33). However, the number of severe cases has notably decreased in comparison to patients who suffer from Delta disease. Although Omicron infections are generally associated with mild symptoms, the mortality rate among infected individuals is approximately 7 to 8 times greater than that of individuals infected with seasonal flu. In particular, the mortality rate was greater than 10% in elderly individuals aged >80 years, which is approximately 100 times greater than the mortality rate associated with the common flu.

It seems that the antibodies developed against previous forms of SARS-CoV-2 are less effective against Omicron variants, therefore, Omicron variants can evade the immune barriers established by prior vaccination, leading to reinfection. According to computational prediction, the risk of reinfection induced by Omicron variants is approximately three times greater than that induced by the prototype, Beta and Delta variant strains (34). Consequently, the Omicron variant exhibits an extraordinary level of transmissibility (one person can potentially transmit the virus to 9.5 other individuals). One study from France identified 188 (0.7%) cases of reinfection by Omicron variants from November 28, 2021~July 22, 2021, indicating that the time between confirmed primary infections and reinfections with different Omicron subvariants is frequently shorter than the 90-day definition of reinfections used by the UCDC and Prevention (35). Starting in May 2022, several countries, including the United States, South Korea and China, experienced a significant surge of pediatric COVID-19 cases, primarily attributed to the Omicron BA.2 lineage (36, 37). On May 4, 2022, the WHO issued a warning, highlighting the need for monitoring of the BA.2.12.1 sublineage, which exhibited a 23%~27% greater transmission rate than BA.2 (38). The combination of the Omicron and Delta variants has given Omicron a transmission advantage. As of June 18, 2022, a report from King’s College London indicated that 2 million individuals were experiencing sequelae of COVID-19 pneumonia, 31.0% of which occurred during the Omicron pandemic in the UK (39).

Recently, the most common COVID-19 cases were reported due to three new SARS-CoV-2 Omicron variants, EG.5 (Eris), FL.1.5.1 (Fornax) and XBB.1.16 (Arcturus), which are common in most countries worldwide (40). EG.5 (Eris) was first reported by the WHO in February 2023 and was designated as a variant under monitoring (VUM) in July. EG.5 (Eris) and its sublineages EG.5.1, EG.5.1.1, and EG.5.2 are close subvariant descendent lineages of the XBB.1.9.2 subvariant. However, compared with the parent subvariant, EG.5 (Eris) has two crucial spike mutations, F456L and Q52H (41). Based on the currently available data, these three new subvariants have similar features to the currently circulating Omicron variant, and mutations in the virus genome can cause increased transmissibility, morbidity, and mortality due to a reduction in vaccine efficiency (42). At the end of 2023, the WHO added a new COVID-19 strain, JN.1, to its list of VOIs. JN.1 was first detected in 12 countries in September 2023, with the highest proportions occurring in Canada, France, Singapore, Sweden, the UK, and the US. Data from the US CDC show that JN.1 is the fastest growing Omicron variant in the US and is responsible for 15~29% of new infections (43).

## 5 The potential immune escape mechanisms of Omicron variants

The host antiviral defenses primarily depend on B cells, which produce virus-specific neutralizing antibodies (nAbs) to fight for viruses, and T cells, which include CD4^+^ T cells that secrete cytokines and CD8^+^ T cells that directly kill the infected virus. To date, eight COVID-19 vaccines, including inactivated whole-virus vaccines (coronavac, BBIBP-CorV, and covaxin), mRNA vaccines (BNT162b2 and mRNA-1273), and adenovirus vector vaccines (Ad26.COV2. S, AZD1222, and Covishield), have been granted emergency approval for use by the FDA (44). However, Omicron variants still cause reinfection, suggesting widespread immune evasion (45).

In general, Omicron variants might utilize three main immune strategies to escape host immune surveillance: ① disturbance of the humoral immune response; ② interruption of the cellular immune response; and ③ disruption of innate immune responses, such as inducing cytokine storms and augmenting apoptosis-related proteins (46). For example, the BA.2.12.1, BA.4 and BA.5 variants bear lineage-specific L452Q/R mutations that cause significant humoral immune escape (47). Han et al. reported that Omicron variants can escape 85% of distinct epitopes from vaccine‐induced serum (48). Live virus neutralization experiments also revealed no neutralizing activity of Omicron variants in some serum samples from recovering COVID-19 patients or vaccine recipients (49, 50), suggesting that Omicron variants significantly promote immune evasion. We systematically summarize the potential immune escape mechanisms of Omicron mutants and discuss them as follows.

### 5.1 The mutation reduces the neutralization of Omicron variants by nAbs

The preliminary step for viral entry was spike protein binding to the ACE2 receptor. Therefore, neutralizing antibodies targeting the RBD can block viral entry by interrupting RBD/ACE2 recognition. Moreover, FcRs can also mediate antibody-dependent cellular cytotoxicity (ADCC) to eliminate infected host cells. Thus, many neutralizing antibodies against the spike protein have been developed as potential therapeutic agents against SARS-CoV-2 infection. Currently, nine neutralizing monoclonal antibodies, including Casirivimab (REGN10933; developer: Regeneron); Bamlanivimab (LY-CoV555; developer: Eli Lilly); Cilgavimab (AZD1061/COV2-2130; developer: AstraZeneca); Etesevimab (LY-CoV016; developer: Eli Lilly); Imidvimab (REGN10987; developer: Regeneron); Regdanvimab (CT-P59; developer: Celltrion); Tixagevimab (AZD8895/COV2-2196; developer: AstraZeneca); Sotrovimab (VIR-7831; developer: GSK and Vir); and Bebtelovimab (LY-CoV1404; developer: Eli Lilly), targeting the SARS-CoV-2 spike protein, are issued with emergency use authorization (EUA) by the FDA for the treatment of mild-to-moderate SARS-CoV-2-infected individuals (51, 52). However, Omicron and its subvariants contain a large number of mutations in the RBD. Conversely, most SARS-CoV-2 vaccines and mAbs are designed to target the S-protein, and it is reasonable to suggest that Omicron variants (BA.2, BA.2.12.1, BA.4/5, and BA.2.75) will escape from preexisting immunity induced by previous natural infection, vaccines, or therapeutic mAbs (53). For example, the D614G mutation in the S-protein increases viral infectivity in susceptible cells by 8- to 10-fold, and both the infectivity and transmissibility of the D614G mutant virus are significantly elevated in a hamster model (54). Therefore, two doses of mRNA vaccines exhibit weaker neutralization of Omicron variants, while a third dose enhances immunity and exacerbates protective effects. Bloom J et al. reported that the K417N and N501Y mutations in the RBD of the S-protein are well tolerated or even enhance ACE2 binding by contributing to humoral immune escape and increased infectivity (55, 56). Compared with those of Delta variants, structural analyses revealed that mutations at E484 and Q493 in Omicron’s RBD receptor-binding motif (RBM) play crucial roles in immune escape (48).

Recently, Güttler T et al. reported three significant mutations (E484K, K417N/T and L452R) responsible for different categories of humoral escape. The K417N/T mutation, which is present in the P.1 and B.1.351 lineages, accounts for class 1 antibody escape. E484K, which is in the B.1.526 lineage, P.1 lineage, P.2 lineage and B.1.351 lineage, accounts for class 2 antibody escape. L452R, which is observed in the B.1.617 and B.1.427/429 lineages, accounts for class 3 antibody escape (57). Screaton G et al. studied the neutralization of Omicron by a large panel of sera collected from convalescents infected with alpha-, beta-, gamma-, or delta Omicron, together with vaccinees administered three doses of the Oxford/AstraZeneca (AZD1222) or the Pfizer BioNtech (BNT16b2) vaccine; they found that Omicron escapes neutralization by the majority of potent mAbs arising after both the early pandemic and infection with the beta variant (45). S477N and E484A showed high resistance to multiple mAbs in neutralization assays (58). Other mutations that confer the ability to escape antibody neutralizing activities in the Omicron variant spike protein include Q493R and G446S, which affect the neutralizing activities of mAbs as well as polyclonal sera (59). These combined data suggest that different mutations in the RBD (K417N, G446S, E484A, Q493R, G496S, Q498R, and N501Y) might prevent the binding of diverse classes of antibodies.

### 5.2 Mutants are unable to activate T cells against Omicron variants

T-cell responses play a pivotal role in combating SARS-CoV-2 infections. Early studies revealed that antigen-specific CD4^+^ T cells induced by vaccination are positively associated with the generation of humoral and CD8^ + ^T-cell responses against SARS-CoV-2 infection (60). Some immunodominant CTLs targeting specific viral epitopes, such as S_269-277_ and Orf1ab_3183-3191_, were identified in HLA-A02:01-positive COVID-19 patients (61). The NP_105-113_-B07:02-specific CTLs efficiently clear various strains, including the original, Alpha, Beta, Gamma, and Delta variants (62). Additionally, S_269~277-_ and NSP3_819~828_-specific CTLs were observed in 81% of HLA-A02:01 and 83% of HLA-A01:01 COVID-19 convalescent individuals (63). Recent research revealed that volunteers vaccinated with the Johnson & Johnson Ad26.COV2. The S-cell adenovirus vector vaccine or the Pfizer BNT162b2 mRNA vaccine exhibited robust and enduring S-protein-specific CD8^+^ and CD4^+^ T-cell responses, conferring cross-protection against Delta and Omicron variants (64). A recent study showed that immunization with the XBB.1.5 Spike vaccine can effectively stimulate T-cell responses against XBB. 1.5 and BA.5, suggesting its potential to activate general cellular immunity against Omicron variants. On the other hand, other vaccines targeting early Omicron strains (spike-BA.5, spike-BF.7, and spike-BQ.1.1) failed to elicit cytotoxic CD8^+^ T-cell responses against XBB.1.5. These findings suggest that the XBB.1.5 subvariant strain may evade the original humoral and cellular immunity, while the Spike-XBB.1.5 protein vaccine can effectively trigger this immune response. Therefore, the development of second-generation vaccines capable of inducing broad-spectrum cellular and humoral immunity is crucial for mitigating the potential impact of viral mutations (65).

Although certain amino acid mutations in Omicron variants are associated with antibody evasion, it remains unclear whether viruses can escape T-cell immune surveillance through mutations. Obermair FJ et al. conducted deep sequencing and identified multiple HLA-I-restricted CD8^+^ T-cell epitopes. They confirmed that some amino acid mutations in the Omicron variants led to reduced or ineffective binding of CTL epitopes to specific HLA-I molecules, therefore preventing efficient antigen presentation to T cells by causing recurrent infections (66). The question of whether mutations induce immune evasion lacks definitive confirmation; theoretically, mutations at specific sites could render T cells incapable of recognizing the virus, weakening the immune response against these epitopes and potentially causing wider outbreaks. However, SARS-CoV-2, a large RNA virus, presents a multitude of epitopes for both CD4^+^ and CD8^+^ T-cell responses, with mutated antigenic sites constituting a small fraction of the overall epitope repertoire. Moreover, increased diversity of the T-cell receptor repertoire (TCR repertoire) reduces the likelihood of viral escape. Therefore, new epitopes generated by single amino acid mutations may not be sufficient to collapse the entire immune system, leading to viral escape. Consequently, further evidence is needed to confirm whether amino acid mutations in the Omicron variant indeed impair CTL functionality, rendering them ineffective at virus clearance.

### 5.3 Hybrid immunity cannot antagonize Omicron variant reinfection

The human body establishes an immune defense through “naturally acquired immunity (NAI)” gained from viral infections and “vaccine-acquired immunity (VAI)” acquired through immunization. Early vaccination efforts led to the establishment of VAI in populations. However, countries with high vaccination rates, such as the United States, experienced breakthrough infections and frequent reinfections with variant strains, suggesting that immunity induced solely by vaccines might not effectively counter infections caused by viral variants. Recent research has shown that individuals recovering from COVID-19 and subsequently receiving vaccinations can develop “hybrid immunity”, which enhances neutralizing antibody titers against different variant strains. Consequently, this hybrid immune system might offer better protection against virus invasion than natural infection or vaccination alone (67, 68).

Studies have indicated that “hybrid immunity” induces broad-spectrum neutralizing antibodies against the original strain, Delta, Omicron, and other variant strains. Additionally, “hybrid immunity” elicits cross-reactive CD8^+^ T-cell responses against Omicron variants and enhances the release of IFN-γ and IL-10 by virus-specific CD4^+^ T cells, thereby inhibiting infection-induced inflammation progression (69, 70). Related to studies in which individuals received two sequential doses of vaccines, research by Jolie M et al. demonstrated that “hybrid immunity” developed after two doses of vaccines (BNTb162b and mRNA-1273) in COVID-19 convalescent individuals enhanced CD4^+^ and CD8^+^ T-cell responses against different variants of the virus’s S-protein (71). Notably, “hybrid immunity” also strengthens anti-RBD antibodies against various variants, clearing the virus through Fc-mediated antibody-dependent cellular cytotoxicity (ADCC) (72, 73). Recent studies further revealed that, compared to those of unvaccinated individuals, those with only vaccine-induced immunity or natural infection, or “hybrid immunity”, induce stronger mucosal immunity. Given the nature of SARS-CoV-2 as an acute respiratory virus, enhancing mucosal immunity through “hybrid immunity” may improve the clearance of Omicron variants (74).

However, Catherine J et al. reported that individuals previously infected with the original strain of SARS-CoV-2, even after receiving three doses of the BioNTech mRNA COVID-19 vaccine (BNT162b2), could establish “hybrid immunity” but were unable to prevent infection from the latter B.1.1.529 variant. Specifically, individuals with “hybrid immunity” had significantly fewer neutralizing antibodies against B.1.1.529 than did those who had never been infected with the original strain, indicating that individuals with hybrid immunity did not acquire immune protection against the Omicron variants (75). Unlike the Alpha, Beta, Gamma, and Delta variant series, the Omicron variant series underwent significant changes in amino acid sequence compared to the original Wuhan strain. Most studies on “hybrid immunity” were conducted before the emergence of Omicron variant strains, and these findings might not be applicable to Omicron infections. Hence, while “hybrid immunity” enhances neutralizing antibody capabilities, the prevention of recurrent infections caused by Omic variants is still insufficient.

### 5.4 The “antigenic original sin” supports reinfection by Omicron variants

The “Original Antigenic Sin” (OAS, also called “Immune Imprinting”) describes the phenomenon in which immunity against pathogens is shaped by the host’s first exposure to a related pathogen, this concept explains that when primary immunity is boosted not by a homologous immune agent but by a cross-reactive vaccine, the newly formed antibodies may react better with the primary antigen than with the antigen actually eliciting the response (76). Mechanistically, when different strains of a pathogen infect a host cell, the immune system dedicates most of the response toward recalling the immune effectors used for the original exposure as opposed to generating responses against the new strains, thus resulting in immune evasion. OASs have been observed to be associated with influenza, dengue, human immunodeficiency virus (HIV), and other pathogens, including SARS-CoV-2 (77, 78).

Similar phenomena have been observed in patients with COVID-19. For example, patients who were previously infected with the Wuhan Hu-1 strain failed to boost neutralizing antibody and T-cell responses against Omicron B.1.1.529, revealing a profound imprinting effect (75). Wang Q et al. showed that immune imprinting impairs neutralizing antibody titers for bivalent mRNA vaccination against the SARS-CoV-2 Omicron subvariant BA.5 or BQ (79). Addetia A et al. showed that vaccine-elicited human plasma antibodies reduce neutralizing activity against the Omicron variants BQ.1.1 and XBB.1.5, although the emerged BQ.1.1 and XBB.1.5 variants bind host ACE2 with high affinity and promote membrane fusion more efficiently than earlier Omicron variants (80). Cao Y et al. isolated monoclonal antibodies from individuals who had BA.2 and BA.5 breakthrough infections and reported that Omicron variants, including BQ.1.1.10 (BQ.1.1 + Y144del), BA.4.6.3, XBB and CH.1.1, reduced the diversity of the neutralizing antibody binding sites and increased the proportions of nonneutralizing antibody clones owing to humoral immune imprinting (81). Even after receiving two doses of the mRNA-1273 vaccine, a third dose did not enhance the neutralizing ability of the Omicron variant in rhesus macaques (82). Moreover, the diversity of antibodies generated after breakthrough infections with the BA.5 variant gradually decreases due to OAS (81). Zhang Z et al. also demonstrated that vaccinated individuals primarily exhibit immune memory shared with alpha, beta, gamma, delta, and other evolved mutants due to OAS, making it challenging to induce specific antibody responses against the BA.2 variant (83).

Early studies have also shown that infections with different serotypes of Dengue virus can activate T cells (84). Additionally, some volunteers vaccinated with an H5N1 influenza live attenuated vaccine (pLAIV) exhibited T-cell responses to seasonal influenza rather than H5N1-specific responses (85), indicating that T-cell responses are also influenced by OAS. However, researchers have discovered that in a cohort of patients who suffered from dengue fever in Sri Lanka, infections with different serotypes of dengue virus could induce a broad and efficient protective IFN-γ^+^ CTL response (86, 87). Recently, Kim et al. tracked memory T-cell responses in several vaccinated individuals in Korea who experienced Omicron subvariant breakthrough infection. They confirmed that BNT162b2 vaccination induced memory CD4^+^ and CD8^+^ T cells specific to the BA.4/BA.5 spike virus, even if these individuals had a prior SARS-CoV-2 infection. They identified peptides in the BA.2 spike that were fully conserved in BA.4/BA.5 and later subvariants but absent in the original spike. These findings provide further evidence that breakthrough infection can induce cross-reactive memory T-cell responses that contribute to protection against newly emerging SARS-CoV-2 subvariants (88).

**Figure 2:**
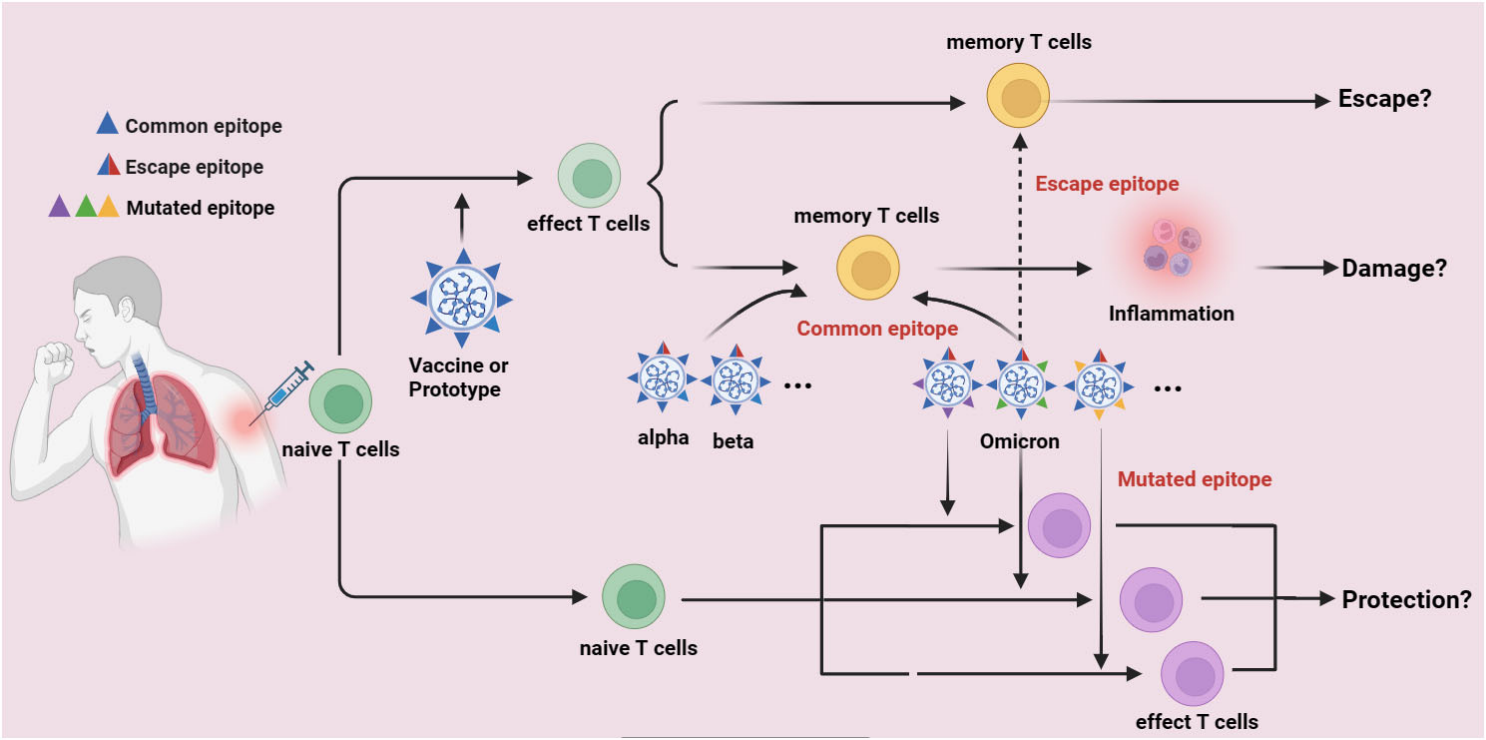
The potential immune escape mechanism of Omicron variants. Lots of immunodominant epitopes are found in the prototype of SARS-CoV-2 (Wuhan-Hu-1) and the first generation of COVID-19 Vaccines which developed are based on the S-glycoprotein of SARS-CoV-2 (common epitopes), these common epitopes can activate naïve T cells and finally differentiate into memory T cells in persons who are suffered from Wuhan-Hu-1 infection or got COVID-19 vaccination. The Omicron variants also have some of these common epitopes, which might also rapidly activate the memory T cells to release inflammatory factors, by thus induce immune damage and immune evasion. However, if the amino acid mutations of Omicron variants are taken place in these common epitopes, these epitopes are possibly unable to activate memory T cells and finally induce immune escape (escape epitopes). Furthermore, if the amino acid mutations are found in some places where leads to the formation of some novel epitopes (mutated epitopes), these mutated epitopes might activate naïve T cells and control Omicron variants spread, thereafter induce immune protection.

Individuals infected with the original SARS-CoV-2 strain or vaccinated against COVID-19 have cross-recognizing CTLs against the B.1.1.7, B.1.351, P.1, and CAL.2C variant sequences. Long-term follow-up studies on 51 mildly or moderately recovered COVID-19 patients showed that certain epitopes could maintain long-lasting T-cell responses. T cells mediating prolonged responses are presumed to protect the body from virus infection. SARS-CoV-2 contains numerous CTL epitopes, therefore, new variants, including Omicron, may not evade T-cell immune recognition. However, the presence of high-frequency virus-specific CTLs in COVID-19 patients does not prevent reinfection by Omicron variants, suggesting that antigens derived from vaccines or the original SARS-CoV-2 strain might constitute the “antigenic original sin”. In fact, overwhelming data have demonstrated that lots of immunodominant epitopes are found in the prototype of SARS-CoV-2 (Wuhan-Hu-1) and the first generation of COVID-19 Vaccines which developed are based on the S-glycoprotein of SARS-CoV-2 (common epitopes), these common epitopes can activate naïve T cells, which are finally differentiated into memory T cells in persons who are suffered from Wuhan-Hu-1 infection or got COVID-19 vaccination. Interestingly, the Omicron variants also have some of these common epitopes, which might also rapidly activate the memory T cells to release inflammatory factors, by thus induce immune damage and immune evasion. However, if the amino acid mutations of Omicron variants are taken place in these common epitopes, these epitopes are possibly unable to activate memory T cells and finally induce immune escape (escape epitopes). Furthermore, if the amino acid mutations are found in some places where leads to the formation of some novel epitopes (mutated epitopes), these mutated epitopes might activate naïve T cells and control Omicron variants spread, thereafter induce immune protection (**Figure 2**).

### 5.5 Other immune evasion mechanisms of Omicron variants

Coronaviruses evade the host immune response by using different mechanisms, which include inhibiting interferon (IFN) communication, antagonizing IFN synthesis, and boosting IFN tolerance. For example, mutations in both ORF8 and NSP6 were associated with increased virulence and antagonism of IFN-I pathways (89, 90). Moreover, the Omicron subvariants BA.4 and BA.5 more potently suppressed innate immunity than did the early subvariant BA.1, which correlated with ORF6 levels (91), suggesting that mutations outside the S-protein affect virus-host interactions and may alter the pathogenesis of SARS-CoV-2 variants in humans. Antigen presentation by major histocompatibility complex class I (MHC-I) cells is a critical step for the activation of antigen-specific CD8^+^ T cells and the subsequent killing of infected cells. To successfully establish infection and replicate in the host, Omicron variants have acquired the ability to inhibit MHC-I processing and the presentation of viral antigens. For example, Omocron variant ORF8 (open reading frame 8) protein induces autophagic degradation of MHC-I and confers resistance to CTL surveillance (92). These findings suggest that during Omicron infection, defective stimulation of MHC-I genetic expression in airways and epithelial cells from the intestines impairs cellular immunity mediated by CD8^+^ T cells (93). Additionally, Omicron variants can now infect target cells *in vitro* without relying on TMPRSS2, suggesting that their replication characteristics have shifted, and this alteration might be one of the reasons behind their immune evasion capabilities (94).

## 6 Perspective

The Omicron variant results in the most mutations, and these mutations are often associated with an extraordinary ability to reinfect and evade immune cells, therefore, Omicron poses a great threat to human public health. To address this challenge, measures such as increasing vaccine coverage, promoting boosters, especially heterologous boosters, maintaining social distancing, and wearing masks could be established to limit the spread of the Omicron variant and reduce the rates of infection and hospitalization.

Understanding Omicron’s immune evasion mechanism and host immune responses, mining other conserved viral epitopes, and improving the immunogenicity of vaccines will all contribute to the design of better vaccines and antibodies against Omicron. Currently, several international research teams are adopting a stepwise approach to extract T-cell epitopes of the SARS-CoV-2 virus S antigen (8~14 amino acids, shifting every 2~3 amino acids). This method involves the use of a peptide library (80~100 peptides) to evaluate T-cell responses within the body of individuals who have received the COVID-19 vaccine or who have recovered from COVID-19 (95). Although this method can overlook HLA differences among individuals, specific information about peptides that can activate T cells remains unclear. Several research teams have utilized epitope peptide binding affinity prediction software, such as netMHC and IEDB (www.iedb.org), to screen SARS-CoV-2 T-cell epitopes. After *in vitro* validation, several immunodominant T-cell epitopes of SARS-CoV-2 were identified (96). For example, Nguyen A et al. reported that HLA-A02:02 and HLA-C12:03 effectively present SARS-CoV-2 epitope peptides (97). This method emphasizes the antigen peptide-HLA binding affinity but lacks strategies for screening peptide immunogenicity.

Recently, our group used a combination of netMHC and PromPPD prediction software to conduct comprehensive genome-wide scanning of CD8^+^ T-cell epitopes for the original strain of the novel coronavirus as well as its variants. We identified immunodominant epitopes based on different HLA-A supertypes. Additionally, we compared the amino acid sequences of the original Wuhan strain and Omicron variant strains, predicting multiple mutation epitopes for various variants (**Supplementary Table S1**). Therefore, by utilizing these identified new epitopes, we can delve into the activation states of virus-specific CTLs within the body at different stages. We can analyze whether different variants possess cross-reactive or dominant epitopes and how memory T cells are regulated by “original antigenic sin”. In this analysis, we elucidated the immune escape mechanism of the Omicron variant.

## 7 Limitations

First, the included studies were time-limited. Omicron variants are rapidly developing, but some recent articles are not cited. Second, many molecular mechanisms of Omicron variant-induced immune escapes have not been explored, it is necessary to continue studying the underlying mechanisms of pandemics caused by Omicron variants, and this information could guide new therapeutic efforts. Finally, further investigations are needed to clarify how “antigenic original sin” supports reinfection by Omicron variants, which could lead to potentially valuable targets for the development of pancoronavirus therapeutics.

## Author contributions

GG: Data curation, Writing – review & editing. LC: Writing – review & editing. YH: Writing – original draft. HL: Writing – original draft. YS: Data curation, Writing – review & editing.

## Supplementary material

Supplementary Table 1 Predicting the immune superiority epitope in S- antigen of different SARS-CoV-2 variants based on netMHC+PromPPD software.
