# Supplementary material for: Potential immune evasion of the severe acute respiratory syndrome coronavirus 2 Omicron variants
Source: Front Immunol. 2024 Feb 23;15:1339660. doi: 10.3389/fimmu.2024.1339660 (PMC10924305; doi:10.3389/fimmu.2024.1339660)
Supplement: Supplementary Table 1 — Predicting the immune superiority epitope in S- antigen of different SARS-CoV-2 variants based on netMHC+PromPPD software. [file Table_1.pdf]

Supplementary Table 1

Table S1 Predicting the immune superiority epitope in S- antigen of different SARS-CoV-2 variants based on netMHC+PromPPD software.

HLA supertype	SARS-CoV-2					
	**Alpha**	**Beta**	**Delta**	**Gamma**	**Omicron**	**Wuhan-1**
A*01	FCNDPFLGVY	FCNDPFLGVY	FCNDPFLDVY	FCNYPFLGVY	FCNDPFLD	FCNDPFLGVY
	QTGKIADYNY	QTGNIADYNY	QTGKIADYNY	QTGTIADYNY	QTGNIADYNY	QTGKIADYNY
	KIADYNYKL	NIADYNYKL	KIADYNYKL	TIADYNYKL	NIADYNYKL	KIADYNYKL
	CNGVEGFNCY	CNGVKGFNCY	CNGVEGFNCY	CNGVKGFNCY	CNGVAGFNCY	CNGVEGFNCY
	NGVEGFNCY	NGVKGFNCY	NGVEGFNCY	NGVKGFNCY	NGVAGFNCY	NGVEGFNCY
	GVEGFNCYF	GVKGFNCYF	GVEGFNCYF	GVKGFNCYF	GVAGFNCYF	GVEGFNCYF
	FQPTYGVGY	FQPTYGVGY	FQPTNGVGY	FQPTYGVGY	FRPTYGVGH	FQPTNGVGY
	PTYGVGYQPY	PTYGVGYQPY	PTNGVGYQPY	PTYGVGYQPY	PTYGVGHQPY	PTNGVGYQPY
	LYQGVNCTEV	LYQGVNCTEV	LYQGVNCTEV	LYQGVNCTEV	LYQGVNCTEV	LYQDVNCTEV
A*02	KIADYNYKL	NIADYNYKL	KIADYNYKL	TIADYNYKL	NIADYNYKL	KIADYNYKL
	EGFNCYFPL	KGFNCYFPL	EGFNCYFPL	KGFNCYFPL	AGFNCYFPL	EGFNCYFPL
	YGFQPTYGV	YGFQPTYGV	YGFQPTNGV	YGFQPTYGV	YSFRPTYGV	YGFQPTNGV
	VLYQGVNCT	VLYQGVNCT	VLYQGVNCT	VLYQGVNCT	VLYQGVNCT	VLYQDVNCT
	YQGVNCTEV	YQGVNCTEV	YQGVNCTEV	YQGVNCTEV	YQGVNCTEV	YQDVNCTEV
	GVNCTEVPV	GVNCTEVPV	GVNCTEVPV	GVNCTEVPV	GVNCTEVPV	DVNCTEVPV
A*24	LYQGVNCTEV	LYQGVNCTEV	LYQGVNCTEV	LYQGVNCTEV	LYQGVNCTEV	LYQDVNCTEV
A*03	SSQCVNLTTR	SSQCVNFTTR	SSQCVNLRTR	SSQCVNFTNR	SSQCVNLTTR	SSQCVNLTTR
	QTGKIADYNY	QTGNIADYNY	QTGKIADYNY	QTGTIADYNY	QTGNIADYNY	QTGKIADYNY
	GFQPTYGVGY	GFQPTYGVGY	GFQPTNGVGY	GFQPTYGVGY	SFRPTYGVGH	GFQPTNGVGY
	TYGVGYQPY	TYGVGYQPY	TNGVGYQPY	TYGVGYQPY	TYGVGHQPY	TNGVGYQPY
	YQTQTNSHR	YQTQTNSPR	YQTQTNSRR	YQTQTNSPR	YQTQTKSHR	YQTQTNSPR
	YQTQTNSHRR	YQTQTNSPRR	YQTQTNSRRR	YQTQTNSPRR	YQTQTKSHRR	YQTQTNSPRR
	QTNSHRRAR	QTNSPRRAR	QTNSRRRAR	QTNSPRRAR	QTKSHRRAR	QTNSPRRAR
B*27	RQIAPGQTGK	RQIAPGQTGN	RQIAPGQTGK	RQIAPGQTGT	RQIAPGQTGN	RQIAPGQTGK
	EGFNCYFPL	KGFNCYFPL	EGFNCYFPL	KGFNCYFPL	AGFNCYFPL	EGFNCYFPL
	FQPTYGVGY	FQPTYGVGY	FQPTNGVGY	FQPTYGVGY	FRPTYGVGH	FQPTNGVGY
	YQTQTNSHR	YQTQTNSPR	YQTQTNSRR	YQTQTNSPR	YQTQTKSHR	YQTQTNSPR
B*44	YQGVNCTEV	YQGVNCTEV	YQGVNCTEV	YQGVNCTEV	YQGVNCTEV	YQDVNCTEV
B*58	VSSQCVNLTT	VSSQCVNFTT	VSSQCVNLRT	VSSQCVNFTN	VSSQCVNLTT	VSSQCVNLTT
	GQTGKIADY	GQTGNIADY	GQTGKIADY	GQTGTIADY	GQTGNIADY	GQTGKIADY
	VGGNYNYLY	VGGNYNYLY	VGGNYNYRY	VGGNYNYLY	VSGNYNYLY	VGGNYNYLY
	GVEGFNCYF	GVKGFNCYF	GVEGFNCYF	GVKGFNCYF	GVAGFNCYF	GVEGFNCYF
B*62	VGGNYNYLY	VGGNYNYLY	VGGNYNYRY	VGGNYNYLY	VSGNYNYLY	VGGNYNYLY
	GVEGFNCYF	GVKGFNCYF	GVEGFNCYF	GVKGFNCYF	GVAGFNCYF	GVEGFNCYF
	YQGVNCTEV	YQGVNCTEV	YQGVNCTEV	YQGVNCTEV	YQGVNCTEV	YQDVNCTEV
B*07	CVNLTTRTQL	CVNFTTRTQL	CVNLRTRTQL	CVNFTNRTQL	CVNLTTRTQL	CVNLTTRTQL
	APGQTGKIA	APGQTGNIA	APGQTGKIA	APGQTGTIA	APGQTGNIA	APGQTGKIA
	APGQTGKIAD	APGQTGNIAD	APGQTGKIAD	APGQTGTIAD	APGQTGNIAD	APGQTGKIAD
	TPCNGVEGF	TPCNGVKGF	KPCNGVEGF	TPCNGVKGF	KPCNGVAGF	TPCNGVEGF
	QPTYGVGYQP	QPTYGVGYQP	QPTNGVGYQP	QPTYGVGYQP	RPTYGVGHQP	QPTNGVGYQP
B*08	CVNLTTRTQL	CVNFTTRTQL	CVNLRTRTQL	CVNFTNRTQL	CVNLTTRTQL	CVNLTTRTQL
	VNLTTRTQL	VNFTTRTQL	VNLRTRTQL	VNFTNRTQL	VNLTTRTQL	VNLTTRTQL
	NSHRRARSV	NSPRRARSV	NSRRRARSV	NSPRRARSV	KSHRRARSV	NSPRRARSV
	SHRRARSVA	SPRRARSVA	SRRRARSVA	SPRRARSVA	SHRRARSVA	SPRRARSVA
